# Therapeutic Immunoadsorption and Conventional Plasma Exchange in ABO-incompatible Renal Transplant: An Exculpatory Evidence

**DOI:** 10.7759/cureus.4787

**Published:** 2019-05-30

**Authors:** Soma Agrawal, Mohit Chowdhry, Raj N Makroo, Sweta Nayak, Shiva P Gajulapalli, Uday K Thakur, Ankit Agrawal

**Affiliations:** 1 Transfusion Medicine, Indraprastha Apollo Hospitals, New Delhi, IND; 2 Transfusion Medicine, Fortis Hospital, Faridabad, IND; 3 Internal Medicine, Saint Peter's University Hospital - Rutgers Robert Wood Johnson Medical School, New Brunswick, USA

**Keywords:** abo incompatible, renal transplant, immunoadsorption

## Abstract

Aim

The objective of this study was to compare the efficacy of immunoadsorption (IA) with conventional therapeutic plasma-exchange (cTPE) in ABO-incompatible (ABOi) renal transplant.

Methods

Data of patients from July 2015 to June 2017 (category-I, number of patients (N) = 11; IA±cTPE) on the average length of stay (ALOS), number of cTPE/IA, antibody-titers (AT), creatinine, patient and graft survival at one year were compared retrospectively with patients in period from February 2012 to June 2015 (category-II, N = 29; cTPE only). AT of patients not decreasing to less than one fold after two cTPE were shifted for IA. For patients undergoing IA, real-time AT was done and IA stopped after target titer (TT <1:8) was achieved. Post-transplant cTPE was done if, titers rebounded to ≥1:8. Intravenous immunoglobulin (IVIG) was given after every cTPE/IA. Cost comparisons were made.

Results

In category-I, seven patients (63.63%) were shifted to IA from cTPE. The mean cTPE procedures in category I and II are 3.5 ± 2.4 and 4.8 ± 2.5, respectively (*p *= 0.206). The mean IA procedures in category-I are 1.6 ± 0.5. The number of patients requiring post-operative TPE was less in category-I than category-II, i.e., *N* = 5, 45.5% vs *N* = 20, 69%, respectively (*p *= 0.171). The expense of IA in category-I vs cTPE in category-II was statistically not significant (*p *= 0.422) but had significant lesser ALOS (*p *= 0.044). Expenses, when a patient undergoes both cTPE and IA (category-I), are significantly higher to category-II (*p *= 0.003). The two groups were comparable in AT, creatinine value, graft and patient survival rates at one year.

Conclusion

Contrary to the general judgment of IA being expensive than cTPE, this study shows equivalent expenditures with comparable therapeutic outcomes.

## Introduction

Significant improvements in immunosuppression and desensitization protocols led to favorable or even comparable patient outcomes between ABO-compatible and ABO-incompatible renal transplants (ABOi) [1, 2]. Among the desensitization protocols for ABOi renal transplants, conventional therapeutic plasma exchanges (cTPE) along with intravenous immunoglobulin (IVIG) has been adequately documented [3].

Immunoadsorption (IA) is a more selective and antigen-specific protocol, that uses columns made of low-molecular-weight carbohydrates with immobilized blood-group A or B antigens linked to a sepharose matrix. These columns specifically deplete anti-B or anti-A antibodies corresponding to the antigen present on it, thereby making it more effective. This protocol has gained traction over the years and is successfully implemented in many well-established centers across the world [4-6]. However, the high cost of IA columns limits their usability in other less-established centers, particularly in developing countries. To overcome this, a modified protocol was recently proposed that allowed the reuse of IA columns without compromising on its efficacy [7-8].

In view of the limited studies documenting the use of this modified protocol, we aimed to compare the efficacy and cost-effectiveness of using goal-directed IA (with the re-use of IA columns wherever needed) over cTPE in achieving the target ABO titers in live related ABOi renal transplants. 

## Materials and methods

In this study, all consecutive patients with chronic kidney disease (CKD) stage IV/V and undergoing ABOi renal transplants at our tertiary care hospital of northern India between 2012 and 2017, were included. All renal transplants were done after obtaining proper informed consent and appropriate histocompatibility workup.

Immunosuppression

**Figure 1:**
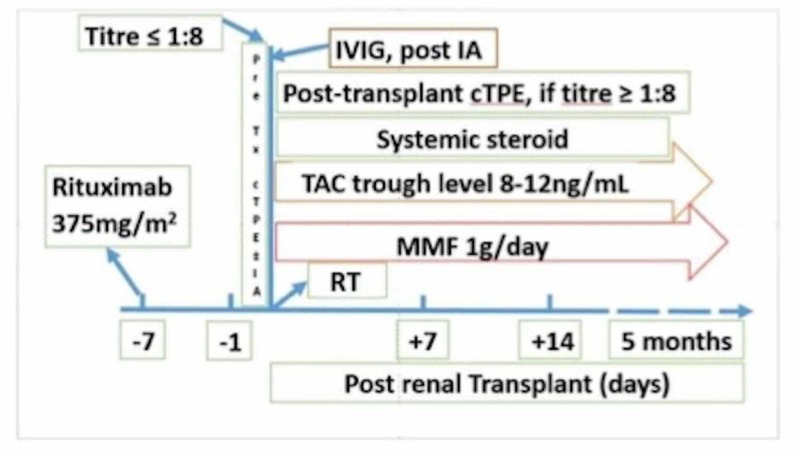
Institutional protocol for ABOi living related renal transplantation TAC: tacrolimus; MMF, mycophenolate mofetil; IVIG, intravenous immunoglobulin; IA, immunoadsorption; RT, renal transplant; cTPE, conventional therapeutic plasma exchange

In all patients, the immunosuppressive protocol consisted of induction with rituximab (375 mg/m^2^) in pre-operative period, followed by tacrolimus (TAC) 0.05 to 0.075 mg/kg twelve hourly and mycophenolate mofetil (MMF) 1 g/day in divided doses, from the day of surgery. Methylprednisolone was started with 1 gm intravenous dose during the intra-operative phase and continued with 100 mg/day and 80 mg/day on post-operative day (POD) one and two followed by switching and tapering with prednisolone. A trough level of 8-12 ng/mL was maintained for TAC in the first month of the post-operative period and tapered after that to 5-8 ng/mL (Figure 1).

Desensitization

Based on the desensitization protocols used, the patients were grouped under two categories namely category I (IA ± cTPE) and category II (cTPE exclusively). cTPE was performed on Hemonetics MCS+ (Braintree, MA, USA). One to one and a half plasma volume was processed per session with five percent albumin as replacement fluid. Two units of group AB, fresh frozen plasma was used as the replacement fluid towards the end of the procedure to maintain the coagulation profile. IA was done using antigen-specific immunoadsorbent column (Glycosorb ABO, Glycorex Transplantation, Lund, Sweden) on Terumo BCT's Spectra Optia®.

Antibody titers

Combined antibody titers (AT; IgM +IgG) were done at the baseline, after each cTPE &/or IA and daily till discharge in both the categories. Polyspecific antihuman globulin (AHG) phase gel card was used for all patients in category I and tube technique was used for all the patients in category II. Though the AT detection methods were different in both the groups, the study focused on the ‘titer reduction' per session of cTPE or IA, rather than the titer technique per se. Moreover, the AT detection method remained the same for all patients pertaining to a single category. For patients undergoing IA, real-time goal-directed AT was done [5] and the procedure was stopped once the target titer (TT < 1:8) was achieved or after processing eight plasma volumes (one IA session), whichever was earlier. In cases the target titers could not be achieved in one IA session, another session of IA was attempted the next day using the same column as described previously [8]. Post-transplant cTPE was done in patients where AT rebounded to ≥ 1: 8 (with or without graft dysfunction) or in patients with elevated serum creatinine levels. In patients where the AT did not decrease (≤ one fold decrease only) with two consecutive procedures by cTPE or IA, they were labeled as “resistant titers” and the procedure was performed using IA or cTPE respectively. IVIG was given in a dose of 0.1 g/kg body weight after every cTPE or IA.

Patient and graft survival

Antibody-mediated rejection (AMR) was assessed on the basis of renal function tests. Kidney biopsies were performed exclusively on the patients who showed features of graft dysfunction. Patient survival was calculated from the date of transplantation to the date of death. Graft survival (non-censored for death) was calculated from the date of transplantation to the date of irreversible graft failure signified by a return to long-term dialysis or re-transplantation or date of death. Death of patient despite having a functional graft was treated as graft failure.

Cost comparison

The average costs for desensitization procedure, IVIG and bed charges/day of tertiary care corporate centers in North India was considered for the cost comparison namely: Mean bed charges/day as 71.52 USD/day (42.91-100.12 USD/day); IVIG cost as 143.03 USD/dose (100.12-185.94 USD/dose); cTPE with replacement fluids as 715 USD/session (500.61-929.69 USD/session), cost for processing eight plasma volumes by using an IA column as 4791.51 USD (4147.87-5435.14 USD) and additional cost of 286.06 USD (214.54-357.57 USD) for the second session of IA using the same column The length of stay was considered the same as the number of preoperative cTPE and/or IA days only for cost comparison purpose.

Statistical analysis

Data on patient demographics, combinations of ABO-incompatibilities, rituximab conditioning, the average length of stay (ALOS); the number of cTPE/IA procedures; antibody titers (AT) at the baseline, transplant and discharge; serum creatinine at discharge, patient and graft survival after one year were prospectively collected for category I and compared with the data of the category II [9]. The ALOS was considered as the mean duration a patient stays in the hospital from the date of admission till discharge.

Tests were done using the statistical package for the social science system version SPSS 20.0 and R-3.2.0. Continuous variables are presented as Mean ± SD (Standard Deviation), and categorical variables are presented as absolute numbers and percentage. Chi-Square test was applied to observe the correlation between categorical variables and T-test was used to compare the differences in continuous variables between category I and II. Z-test of proportion was used to compare the proportion of parameters between the two categories. For all statistical tests, a p-value less than 0.05 was considered clinically significant.

## Results

There were eleven patients (nine males and two females) in the category I with mean age 31.27 ± 13.5 years. Abiding by the set protocol, seven of the eleven patients (two patients with anti-A and five patients with anti-B antibody titer) were started on cTPE and shifted to IA due to the resistant titers. Four of them were directly started on IA in the category I, owing majorly to the patient affordability. One of these four patients was switched to cTPE after IA due to the resistant titer of anti-A antibody. In category II, there were twenty-nine patients. Of them, twenty-three (79.3%) were males and six (20.7%) females with an average age of 39.03 ± 14.53 years. All of these patients underwent cTPE for reduction of AT.

The two categories were comparable in terms of age (*p* = 0.127) and gender distribution (*p* = 0.859). The ABO incompatible donor-recipient combinations in both categories are detailed in Table 1.

**Table 1:** ABO incompatible donor-recipient combinations in category I and II. N, number of patients

Blood group (Donor to Recipient)	Antibody titer monitored	Category I (N, %)	Category II (N, %)
A to O	Anti-A	1, 9.1%	6, 20.7%
B to O	Anti-B	7, 63.6%	7, 24.1%
AB to B	Anti-A	2, 18.2%	3, 10.3%
AB to A	Anti-B	0	3, 10.3%
A to B	Anti-A	1, 9.1%	6, 20.7%
B to A	Anti-B	0	4, 13.8%
AB to O	Anti-A & Anti-B	0	0
Total		11	29

Comparison of the number of procedures; ALOS, AT at baseline, transplant, and discharge; creatinine values; the patient and graft survival in one year are shown in Table 2.

**Table 2:** Comparison of patient data in category I and II SD, standard deviation; cTPE, conventional therapeutic plasma exchange; IA, immunoadsorption; N, number of patients; ALOS, average length of stay; AT, antibody titers

Parameter	Category I	Category II	p value
Pre transplant cTPE procedures (Mean ± SD)	3.5 ± 2.4	4.8 ± 2.5	0.206
IA procedures (Mean ± SD)	1.6±0.5	--	--
Number of patients requiring post-operative cTPE (N, %)	5, 45.5%	20, 69%	0.171
Mean number of post-operative cTPE	2	3.1	0.718
ALOS in days (range)	17.8 (8-27)	26.9 (10-95)	0.044
Median AT at baseline (range)	64 (32 to 1024)	64 (16 to 512)	--
Median AT at transplant (range)	2 (1 to 4)	2 (1 to 16)	--
Median AT at discharge (range)	4 (1 to 16)	4(1 to 128)	--
Serum creatinine at discharge (Mean ± SD)	1.08 ± 0.44	1.34 ± 0.9	0.316
Patient survival at one year	9 (81.8%)	27 (93.1%)	0.289
Graft survival at one year	7 (77.8%)	25 (89.3%)	0.378

In the pre-transplant period, mean cTPE procedures in the category I and II were 3.5 ± 2.4 and 4.8 ± 2.5, respectively (*p* = 0.206). Mean IA procedures in the category I was 1.6 ± 0.5.

The number of patients needing post-operative cTPE was less in the category I than category II i.e. N = 5, 45.5% vs N = 20, 69%, respectively (*p* = 0.171) though it did not reach statistical significance. The two groups were comparable in AT at all times, creatinine value, graft and patient survival rates after one year (Table 2).

The expense of IA in the category I vs cTPE in the category II was statistically not significant (p =0.422) (table 3) but had significant lesser ALOS (*p* = 0.044; Table 2). The expenses when a patient undergoes both cTPE and IA (category I) are significantly higher to only cTPE (category II, *p* = 0.003; Table 3).

**Table 3:** Comparison of expenses in USD (mean cost/patient) between category I and II *Category I expenses calculated considering only IA sessions; **category I expenses calculated when patient undergoing IA sessions after failure of cTPE.

Category I	Category II	p value
5558.66*	5141.67	0.422
8289.23**	5141.67	0.003

Two patients (18.2%) in the category I succumbed to death within the first three weeks post-transplantation owing to fungal granulomas in brain parenchyma and septic shock. Among the survivors, two (22.2%) patients had graft dysfunction. The first patient showed chronic inflammatory cell infiltrates in the interstitium, focal tubulitis, patch areas of moderate interstitial fibrosis with tubular atrophy and on graft biopsy. C4d staining was negative. Overall, the picture is suggestive of acute on chronic rejection. After graft biopsy, the second patient showed patchy fibrosis in the interstitium; atrophic tubules in the scarred area and thick basement membranes. Interstitium had lymphocytic infiltrate which was severe in areas of fibrosis. Mild to moderate lymphocytic tubulitis was seen. C4d staining was seen in 85% of peritubular capillaries. All these features were suggestive of acute cellular rejection in a background of chronic allograft nephropathy.

Two patients (6.9%) in the category II succumbed due to sepsis. Of the survivors, one experienced acute humoral rejection within twenty-four hours of transplant. The second patient faced graft dysfunction within the first week of transplant. Graft biopsy revealed neutrophil infiltrates in the glomeruli and peritubular capillaries. There were tubular injury and C4d staining of 30%. This patient was salvaged with cTPE and other medications. The third patient succumbed due to TMA (diagnosed on the basis of clinical features) related complications in spite of cTPE procedures and supportive drugs. The biopsy described coagulative necrosis and hemorrhage in the cortical tissue with the absence of viable glomeruli. There was patchy interstitial edema, congestion of peritubular capillaries, patchy infiltrates, and hyaline casts, suggestive of acute AMR.

## Discussion

This study presents the comparison between two modalities of antibody titer reduction for ABO-incompatible renal transplant i.e. IA with or without cTPE (category I) and only cTPE (category II). The average number of procedures, ALOS, post-transplant procedures, patient and graft survival after one year of transplant were compared. As per the analysis, the ALOS of the patients in the category I was eighteen days. This was found to be significantly different from that of the category II where the average length was of twenty-seven days. Other parameters like the average number of procedures, procedures performed after the transplant, patient and graft survival after one year of transplant were found comparable among the two groups. The other aspect explored was the finances involved, which has continued to be a major concern for non-insured patients. The study demonstrates a significant difference between the expenditure with patients who were switched over to IA from conventional TPE owing to resistant titers; as compared to the patients who underwent conventional TPE only. However, the expenses for IA only were found comparable to the expenses for undergoing exclusive cTPE.

Schwenger et al. described the various indications for the use of IA columns [10]. However, they could not find any direct comparisons for the use of IA and/or cTPE in ABOi kidney transplant patients in the literature. The present study attempts to address this gap. The ABOi transplant outcomes of in the present study were comparable in terms of graft and patient survival at one year of follow-up either by using cTPE alone or in combination with IA for desensitization. Moreover, the use of IA prepared the patients for transplant well in time with minimal ALOS, thereby improving the quality of life.

A similar comparison between IA and cTPE has also been attempted to assess the AT reductions in ABOi renal transplant by Parmentier et al. [11]. They concluded that though cTPE showed good AT reduction efficacy, to prevent complications arising due to cTPE, the combined use of IA with a membrane filter represents a possible and effective way. Data on such comparative analysis between IA and cTPE are also available in various other clinical settings [12-13].

Tyden et al. discussed outcomes in eleven patients undergoing ABOi renal transplant [14]. The IA and immunosuppression protocol used in the current study were, to a far extent, similar to theirs. They used IA on pre-transplant day −six, −five, −two and −one with the target titer remaining the same as defined in the current study. Each session of IA composed of processing 1.5-2 plasma volumes. Use of IA columns led to promising results with good outcomes of the transplant. Though our results were similar and very much comparable to those described by Tyden et al., the extended use of IA column to process up to eight plasma volumes in one session and re-use of the same column, wherever needed was an important and significant time and cost-saving effort whilst maintaining the efficacy [14]. One more significant finding to notice is there were two patients as quoted by Tyden et al. [14] in their study, whose transplantation was postponed because of resistant titers after four sessions of IA and needed few additional IA. These would ultimately amount to a high expense and long waiting periods for transplantation. However, with the rapid shift to IA from cTPE for the seven patients in the category I, who had resistant titers and the use extended protocol of processing up to eight volumes of plasma with IA, there were no such delays. Of the remaining patients in category I, three were transplanted without any delay after using IA as the desensitization modality. One patient in the category I achieved the required AT only after switching over to cTPE from IA. Though the nature of the antibodies in this patient was not investigated, the circumstances suggest the presence of core chain antibodies which is usually found in 13% of healthy individuals [15]. However, the timely switch over to cTPE due to the resistant titers did not lead to any undue delay in the transplant. 

Alongside these advantages, not using replacement fluids like albumin/fresh frozen plasma (FFP) in IA leads to fewer donor exposures and maintains the immunoglobulin levels.

Koo et al. [16] mention that the antibody reduction with IA results in a similar rate of graft survival compared with cTPE. However, it also reiterates the fact that at least four sessions of IA are needed in the pre-transplant period to achieve the target titers. This is in consensus with that described by Tyden et al. [14]. The authors also state this as the reason of high cost and therefore, not affordable to patients who are uninsured. This highlights the need for strategies to improve affordability for IA whilst maintaining the efficacy and preparing the patient in time for the transplant. Moreover, the variable that requires attention is the length of stay involved in performing the desensitization procedures irrespective of cTPE &/or IA. This study proves the ALOS is decreased by performing goal-directed, extended use of IA columns over cTPE. This outcome is significant as it has a direct impact on not only the overall expenses but also on the quality of life of the patient. Contrary to the notion of IA being expensive, on average, the costs incurred to the patient irrespective of cTPE or IA is not statistically different (p = 0.422). The cost of IA per se which is higher is probably adjusted by the lesser number of days admitted in the hospital, which compensates for the difference. However, if the patient is started on cTPE and shifted to IA protocol due to resistant titers, the expenses are significantly higher (*p* = 0.003).

As explained in another study by Tyden et al. [17], the early rebound of antibody titers post-transplantation was noticed in four out of eleven (36.4%) patients requiring post-operative adsorptions. However, there was no late rebound of antibodies in their study. Similarly, in our study, the number of patients requiring early post-operative cTPE in the category I (45.5%) was less as compared to that in category II (69%). Also, it was comparable to that described by Tyden et al. [17]. Although 45.5% of the patients needed a post-transplant cTPE, the number of procedures required was very less i.e. mean of two procedures per patient. None of them in the current study had a late rebound of antibodies nor needed any cTPE procedures until follow-up of one year which is also supported by Tyden et al. [17]. By contrast, the number of patients with the early rebound of antibodies in category II (69%) was higher than in the category I, though not statistically significant. These were comparable to those described by Tobian et al. (76.9%) and there were no late rebounds in this category as well [18].

The median titers in both the groups before the transplant, at transplant and discharge were comparable. The serum creatinine values, patient and graft survival were comparable among the groups showing the clinical efficacy of either of the procedures implemented are similar.

In the present study, two and one patient from the category I & II respectively succumbed due to infection and sepsis. There have been conflicting and inconsistent findings with regard to infectious rates with or without rituximab and plasma exchange desensitization regimens. Some of them support the finding of higher overall infection rate in ABOi transplants as compared to that of ABO-compatible transplants whereas others contradict [19-23].

Our study was not without limitations. To start with, comparison for the number of the fold of decrease in antibody titer by IA alone vs cTPE was not performed because of a statistically limited sample size number (N = 4) for patients undergoing only IA without prior cTPE. The complications and infection rate related to the ALOS in both categories were not evaluated. The choice of IA was dependent solely on patient affordability. Lastly, a comparison between the equipment used for cTPE and IA was not considered.

## Conclusions

The efficacy of the goal-directed extensive use of IA column was as efficacious as the cTPE. Moreover, it has an added advantage of decreased ALOS, thereby cutting down the costs and making it an affordable approach and improving the quality of life of the patients.
